# Black Carbon Exposure, Oxidative Stress Genes, and Blood Pressure in a Repeated-Measures Study

**DOI:** 10.1289/ehp.0900591

**Published:** 2009-07-31

**Authors:** Irina Mordukhovich, Elissa Wilker, Helen Suh, Robert Wright, David Sparrow, Pantel S. Vokonas, Joel Schwartz

**Affiliations:** 1 Department of Epidemiology, University of North Carolina–Chapel Hill, Chapel Hill, North Carolina, USA; 2 Department of Environmental Health, Harvard School of Public Health, Boston, Massachusetts, USA; 3 Channing Laboratory, Brigham and Women’s Hospital, Boston, Massachusetts, USA; 4 Veterans Administration Normative Aging Study, Veterans Affairs Boston Healthcare System and the Department of Medicine, Boston University School of Medicine, Boston, Massachusetts, USA

**Keywords:** black carbon, blood pressure, epidemiology, gene–environment interactions, human, oxidative stress, particulate matter

## Abstract

**Background:**

Particulate matter (PM) air pollution has been associated with cardiovascular morbidity and mortality, and elevated blood pressure (BP) is a known risk factor for cardiovascular disease. A small number of studies have investigated the relationship between PM and BP and found mixed results. Evidence suggests that traffic-related air pollution contributes significantly to PM-related cardiovascular effects.

**Objectives:**

We hypothesized that black carbon (BC), a traffic-related combustion by-product, would be more strongly associated with BP than would fine PM [aerodynamic diameter ≤ 2.5 μm (PM_2.5_)], a heterogeneous PM mixture, and that these effects would be larger among participants with genetic variants associated with impaired antioxidative defense.

**Methods:**

We performed a repeated-measures analysis in elderly men to analyze associations between PM_2.5_ and BC exposure and BP using mixed-effects models with random intercepts, adjusting for potential confounders. We also examined statistical interaction between BC and genetic variants related to oxidative stress defense: *GSTM1*, *GSTP1*, *GSTT1*, *NQO1*, catalase, and *HMOX-1*.

**Results:**

A 1-SD increase in BC concentration was associated with a 1.5-mmHg increase in systolic BP [95% confidence interval (CI), 0.1–2.8] and a 0.9-mmHg increase in diastolic BP (95% CI, 0.2–1.6). We observed no evidence of statistical interaction between BC and any of the genetic variants examined and found no association between PM_2.5_ and BP.

**Conclusions:**

We observed positive associations between BP and BC, but not between BP and PM_2.5_, and found no evidence of effect modification of the association between BC and BP by gene variants related to antioxidative defense.

Cardiovascular morbidity and mortality have been associated with particulate matter (PM) air pollution in numerous epidemiologic studies (Brook 2008; Miller et al. 2007; Pope et al. 2004). Several pathways have been proposed to explain these associations, including, at the molecular level, increased oxidative stress (Gurgueira et al. 2002; Kim et al. 2004; Nel 2005) and systemic inflammation (Chuang et al. 2007; Dubowsky et al. 2006; O’Neill et al. 2007; Rückerl et al. 2006). At the functional level, potential pathways include changes in autonomic function (Devlin et al. 2003; Gold et al. 2005; Park et al. 2005) and in blood pressure (BP) (Urch et al. 2005; Zanobetti et al. 2004). Elevated BP is a major risk factor for coronary heart disease and stroke. To date, only a few studies have examined the role of PM air pollution in BP elevation, and these have revealed both positive (Auchincloss et al. 2008; Choi et al. 2007; Ibald-Mulli et al. 2001; Zanobetti et al. 2004) and negative (Harrabi et al. 2006; Ibald-Mulli et al. 2004; Madsen et al. 2006) findings.

The heterogeneous composition of PM, which includes a complex mixture of particles and liquid droplets consisting of organic chemicals, acids, metals, and dust particles, may explain some of the variation observed in these studies as a result of differences in PM composition across geographic regions (Brook 2008). Growing evidence suggests that traffic-related PM pollution contributes significantly to PM-related cardiovascular effects (Laden et al. 2000; Schwartz et al. 2005; Zanobetti and Schwartz 2006).

Black carbon (BC) is a traffic-related particle produced as a combustion by-product and has been associated more strongly than has fine PM [aerodynamic diameter ≤ 2.5 μm (PM_2.5_)] with a number of cardiovascular end points (Dockery et al. 2005a, 2005b; Gold et al. 2005; O’Neill et al. 2005; Schwartz et al. 2005). BC is also associated with increased risk of emergency myocardial infarction hospitalization (Zanobetti and Schwartz 2006). No studies conducted among healthy persons have previously assessed the relation between BC and BP outcomes.

We hypothesized that BC would elevate BP in our cohort and that this PM component may in part explain the mixed results observed in the association between BP and PM_2.5_. Because PM air pollution may elevate BP by increasing oxidative stress (Brook 2008; Sørensen et al. 2003), we also hypothesized that the effects of BC on BP would be stronger among participants with impaired anti-oxidative defense. Therefore, we examined the associations of BC and PM_2.5_ with BP in a repeated-measures analysis and investigated the possibility of effect modification of the association between BC and BP by gene variants related to oxidative stress defense.

## Materials and Methods

### Study population

Our study participants were from the Veterans Administration Normative Aging Study. This is an ongoing longitudinal study of aging established in 1963, details of which have been published previously (Bell et al. 1972). Briefly, the Normative Aging Study is a closed cohort of 2,280 male volunteers from the Greater Boston, Massachusetts, area who were 21–80 years of age at entry and who enrolled after an initial health screening determined that they were free of known chronic medical conditions. Participants were reevaluated every 3–5 years using detailed on-site physical examinations and questionnaires. BP was measured in all participants still presenting for examination between April 1999 and December 2007 (791 participants, 1,568 clinical examinations). Some of these participants presented only once during this time period, and our analysis was restricted to 461 participants (1,067 clinical examinations) with complete information regarding ambient BC concentrations and all covariates for at least two study visits, and a largely overlapping group of 457 participants (949 clinical examinations) with complete information regarding ambient PM_2.5_ concentrations and all covariates for at least two study visits. This study was reviewed and approved by the institutional review boards of participating institutions. All participants provided written informed consent.

### Physical parameters and medical history

Study center visits were conducted in the morning after an overnight fast and abstention from smoking. Physical examinations included measurement of height and weight, and body mass index was calculated as [weight (kilograms)/height (meters) squared]. Participants were classified as having diabetes based on a physician’s diagnosis of type 2 diabetes and/or use of diabetes medications. Questionnaires collected details on smoking habits and medication use, with responses confirmed by an on-site physician. Alcohol intake was determined using a semiquantitative food frequency questionnaire. Serum fasting glucose concentrations were measured using the hexokinase method, with measurements performed in duplicate on an auto-analyzer (Leon et al. 1977).

### BP measurements

At each clinical visit, a physician measured BP using a standard mercury sphygmomanometer with a 14-cm cuff. Systolic blood pressure (SBP) and fifth-phase diastolic blood pressure (DBP) were measured in each arm to the nearest 2 mmHg while the participant was seated. The means of the right and left arm measurements were used as each participant’s systolic and diastolic BP. Although there was no specific rest period before measurement of BP, SBP and DBP were measured immediately after a complete patient history was taken with the subject seated.

### Genotyping

#### GSTM1

Genotyping for the glutathione *S*-transferase mu-1 (*GSTM1*) deletion polymorphism (UniGene Hs.301961; UniGene 2009a) was done at the Harvard School of Public Health. The assay consists of polymerase chain reaction (PCR) amplification of exons 4 and 5 of the *GSTM1* allele. Because this polymorphism is a gene deletion, the PCR product indicates the presence of one or more copies of the gene. In each case, concomitant amplification of the cytochrome P450 1A1 gene (*CYP1A1*) (UniGene Hs.72912; UniGene 2009b) was done as a positive control. The PCR amplification of *CYP1A1* results in a 312 base-pair product that is easily visualized in the presence or absence of the *GSTM1* 273 base-pair PCR product. The DNA template was prepared by mixing whole blood (0.5–1.0 μL) with reaction buffer and primers, followed by incubation. To this, 2.5 U Amplitaq was added; PCR was then performed, yielding the 273 base-pair product.

#### GSTP1

We genotyped two single nucleotide polymorphisms (SNPs) on the glutathione *S*-transferase pi-1 gene (*GSTP1*) (UniGene Hs.523836; UniGene 2009c): Ile105Val (A313G, rs1695), caused by an A-to-G substitution that changes codon 105 from ATC (Ile) to GTC (Val); and Ala114Val (C2293T, rs1799811), a C-to-T substitution that changes codon 114 from GCG (Ala) to GTG (Val) (Ali-Osman et al. 1997). Both SNPs were genotyped using unlabeled mini-sequencing reactions and mass spectrometry via a Sequenom Mass Array MALDI-TOF (matrix-assisted laser desorption ionization time-of-flight) mass spectrometer (Sequenom, San Diego, CA) (Sun et al. 2000).

### *HMOX-1* (GT)_n_ repeat polymorphism

The heme oxygenase-1 (*HMOX-1*; UniGene Hs.517581; UniGene 2009d) short-tandem-repeat assay was designed as described by Yamada et al. (2000). Resulting PCR products were analyzed with a laser-based automated DNA sequencer, and the sizes were compared with the human genome sequence (http://genome.ucsc.edu/) to determine the number of repeats.

### *NQO1* and catalase

SNPs within the catalase gene (UniGene Hs.502302; UniGene 2009e) [C/T (rs480575); C1167T (rs769217); C(–262)T (rs1001179); A/G (rs2284367); and A/G (rs2300181)] and NAD(P)H quinine oxidoreductase 1 gene (*NQO1*; UniGene Hs.406515; UniGene 2009f) [C609T (rs1800566)] were genotyped using the Sequenom MassArray MALDI-TOF mass spectrometer (Sequenom) with semiautomated primer design (SpectroDESIGNER, Sequenom).

### GSTT1

Analyses were performed at the Harvard School of Public Health using the TaqMan 5′ nuclease real-time quantitative PCR assay. Subjects were genotyped for glutathione *S*-transferase theta-1 gene (*GSTT1*) deletions (UniGene Hs.268573; UniGene 2009g). For this analysis, the following primers and probe were used: *GSTT1* exon 1 forward primer (5′-caggtcagcacttaagcgatgc-3′), *GSTT1* reverse primer (5′-cagcagtcctaggg tcccagtta-3′), and *GSTT1* probe (5′-Famccttcaaggctggcaccttcttgagg-Tamra-3′).

### BC, PM_2.5_, and meteorologic measurements

Continuous BC and PM_2.5_ concentrations were measured at a Harvard School of Public Health monitoring site located at the Countway Library (Boston, MA), 1 km from the clinical examination site, and were averaged by hour before BP measurement. BC was measured using an aethalometer (Magee Scientific, Berkeley, CA), and PM_2.5_ was measured using a tapered element oscillating microbalance (model 1400A; Rupprecht & Pataschnick Co., East Greenbush, NY), operated at 50°C with two 4-L/min PM_2.5_ impactors before the inlet. We obtained temperature and relative humidity measures from the Boston airport weather station.

### Statistical methods

We analyzed associations between both BC and PM_2.5_ exposures and BP in a repeated-measures study using linear mixed-effects models with random intercepts and a block-diagonal (compound symmetry) covariance structure. In this approach, individual-level covariates are allowed to explain differences across subjects. In addition, a separate random intercept is fitted for each subject, so essentially all differences across subjects are controlled and the estimates of association reported are effectively from within-subject differences.

Previous studies have suggested that longer averaging times are more relevant to the association between PM and BP (Zanobetti et al. 2004), so we used 7-day moving averages of ambient BC and PM_2.5_ concentrations matched on the time of BP measurement for each participant. As a sensitivity analysis, we also evaluated the relation between PM and BP using averaging times ranging from 1 hr to 7 days before BP measurement. We evaluated SBP and DBP as dependent variables.

The following covariates were chosen *a priori* and included in the regression models regardless of statistical significance: age (years), cigarette smoking (never, current, or former), pack-years of smoking, body mass index (kilograms per square meter), alcohol intake (< 2 drinks per day vs. ≥ 2 drinks per day), fasting glucose (millimoles), current statin use (yes/no), diabetes status (yes/no), race/ethnicity, years of education, hour of clinical visit, year of clinical visit, weekday of clinical visit, and season of clinical visit (spring: March, April, May; summer: June, July, August; fall: September, October, November; winter: December, January, February). We also included temperature (degrees Celsius; 7-day moving average) and relative humidity (percent; 7-day moving average) in our models *a priori*, along with their quadratic terms. Both temperature and relative humidity variables were centered on their means.

We identified current antihypertensive medication use (yes/no) as a potential confounder *a priori*. Because these medications may be protective for the association between PM air pollution and BP, we also tested for interactions with the pollutants before entering the medication variable into models to control for confounding. In addition, we conducted a sensitivity analysis to examine the effect of including antihypertensive medication use in our regression models because of concern about adjusting for a potential downstream consequence of the BP outcome. Exclusion of this variable did not appreciably alter results (data not shown). After weighing the evidence, we decided to include a term for current antihypertensive medication use in all regressions.

We also examined effect modification of the association between BC and BP by gene variants related to oxidative stress defense, because of prior research findings regarding mechanisms mediating the hypertensive effect of PM air pollution (Brook 2008; Sørensen et al. 2003). We examined polymorphisms in the following antioxidative-defense–related genes: *GSTM1*, *GSTP1*, *GSTT1*, *HMOX-1*, *NQO1*, and catalase.

To assess effect modification by *GSTM1* and *GSTT1* genotypes (wild type vs. null) and by *HMOX-1* microsatellite (GT)_n_ repeat length (< 25 repeats vs. ≥ 25 repeats), we modeled separate regressions stratified by genotype and also added interaction terms between BC concentration and each genotype to respective main effects models. To assess effect modification by SNPs, we first aggregated information regarding each SNP and classified the genotypes in two different manners: categorical (wild-type vs. any variant allele) and continuous (0, 1, or 2 variant alleles). For both of these classifications, we modeled separate regressions stratified by genotype and also added an interaction term between BC and the aforementioned variables to respective main effects models.

We conducted a simulation study to analyze our power to detect gene–environment interactions. We generated repeated measures of exposure as multivariate normal with a correlation of 0.2 across multiple measurements, which were separated by approximately 4 years on average in our cohort. We then generated random binomial data to represent genotype, varying prevalence rates and the interaction between exposure and genotype, and generated the outcome to be multivariate normal with a higher correlation between repeated measures (0.5). We took the main effect of exposure to be the average of the effect we found for DBP and SBP, and the variance of the outcome to be the mean of the variances of the two BP measures. We then performed a regression on the data and calculated the *t*-statistic for the interaction. Finally, we repeated this analysis 1,000 times for relative effect modification of 2 and 3 and took the power to be the fraction of time a significant interaction was found.

## Results

### Study population

Our study population consisted entirely of males, most of whom were former cigarette smokers and current antihypertensive medication users at their first study center visit (Table 1). At this initial examination, participants evaluated for the association between BC and BP had a mean age of 71 years, a mean body mass index of 28 kg/m^2^, and average SBP and DBP of 132 mmHg and 78 mmHg, respectively. Participants evaluated for the association between PM_2.5_ and BP had a mean age of 72 years, a mean body mass index of 28 kg/m^2^, and average SBP and DBP of 131 mmHg and 76 mmHg, respectively. In both of these groups, we averaged pollutant concentrations across all study center visits and found that mean BC concentration was 1.1 μg/m^3^ and mean PM_2.5_ concentration was 12 μg/m^3^.

### Associations of BC and PM_2.5_ concentrations with BP

We evaluated the associations of SBP and DBP with both ambient BC and PM_2.5_ concentrations and express the results as the change associated with a 1-SD (equivalent to 0.43 μg/m^3^ for BC and 4.98 μg/m^3^ for PM_2.5_) increment in exposure to each pollutant (Table 2). We found that a 1-SD increase in BC concentration was associated with a 1.46-mmHg increase in SBP [95% confidence interval (CI), 0.10–2.82; *p* = 0.0356] and a 0.87-mmHg increase in DBP (95% CI, 0.15–1.59; *p* = 0.0180). In contrast, we did not find an association between PM_2.5_ and either SBP or DBP (Table 2). For BC, associations were strongest using a 7-day moving average, and associations between PM_2.5_ and BP remained close to the null for all averaging times examined (data not shown).

### Effect modification by SNPs in oxidative stress genes

We observed no evidence of statistically significant interaction between BC concentration and any of the gene variants assessed (Tables 3 and 4) using either categorical or continuous classifications of SNPs.

### Effect modification by antihypertensive medication use

We found no evidence of statistical interaction between PM_2.5_ and antihypertensive medication use (data not shown). Similarly, stratified estimates for the effects of BC on BP differed very little by use of antihypertensive medications and did not show a consistent difference for SBP and DBP (data not shown).

### Power to detect gene–environment interactions

Our simulation study showed that, for a relative effect modification of 2, we had 47% power for a gene prevalence of 20% rising to 67% power for a gene prevalence of 50%. However, for a relative effect modification of 3, we had 81% power even with a gene prevalence of 20%.

## Discussion

We found that BC concentration averaged over the 7 days preceding each study center visit was positively associated with SBP and DBP in a cohort of elderly men. We did not observe an association between a 7-day moving average of PM_2.5_ and either SBP or DBP and did not find evidence of effect modification of the association between BC and BP by genotypes related to oxidative stress defense. Ours is one of few studies to assess the relation between PM air pollution and BP among participants otherwise healthy at baseline. BC is a traffic-related particle, and our findings of a relation between BC and BP, if confirmed, may have important implications because of widespread exposure to traffic emissions across the U.S. population.

A panel study of 16 elderly subjects with respiratory disease did not reveal an association between BC and BP (Jansen et al. 2005). Furthermore, although a repeated-measures analysis of 62 cardiac rehabilitation patients showed that 120-hr averages of ambient BC were positively associated with resting DBP in single-pollutant models, this association was found to be confounded by PM_2.5_ (Zanobetti et al. 2004). A randomized trial of an improved cook stove among Guatemalan women showed that the use of chimney woodstoves, which resulted in lower personal PM exposures, was associated with a reduction in SBP and DBP relative to a traditional open-wood-fire control group (McCracken et al. 2007). Wood smoke is an important source of BC exposure.

The literature regarding the relation between PM and BP is inconsistent. Epidemiologic studies have shown positive (Auchincloss et al. 2008; Choi et al. 2007; Dvonch et al. 2009; Ibald-Mulli et al. 2001; Zanobetti et al. 2004), null (Jansen et al. 2005; Madsen et al. 2006), and inverse associations (Harrabi et al. 2006; Ibald-Mulli et al. 2004) between PM and BP end points, and the results of chamber studies have been inconsistent, as well (Brook et al. 2002; Urch et al. 2005). Auchincloss et al. (2008) report associations between PM_2.5_ and BP only in areas with a high impact from traffic. Similarly, Dvonch et al. (2009) found much stronger associations between PM_2.5_ and BP in an area with high traffic density relative to other sections of the same city. Discrepant findings between investigations may be attributable to differences in study design and population susceptibility, as well as varying composition of PM across study sites. In particular, investigations include cross-sectional studies that are more susceptible to confounding by factors varying across subjects, panel studies with small numbers of subjects but many repeated measures, and those like the present study, with a smaller number of repeated measures in a larger cohort.

Research regarding mechanisms mediating BC’s hypertensive effects is quite limited. Plasma markers of systemic oxidative stress and inflammation have been associated with BC exposure in epidemiologic studies (Delfino et al. 2008; Sørensen et al. 2003). However, much of our discussion regarding potential mechanisms of BC cardiotoxicity is informed by broader PM research. Our results suggest that BC may be primarily responsible for the association of PM with BP.

In addition to examining effects of BC on BP, we also hypothesized that these effects would be stronger among participants with genetic variants associated with impaired oxidative stress defense and therefore evaluated statistical interactions between BC exposure and relevant gene variants. We did not find statistically significant evidence of effect modification by antioxidative defense genes in this analysis. Although our findings may suggest that the effect of BC on BP is not mediated by the oxidative stress–related mechanisms associated with the examined genetic variants, this analysis may not have had sufficient power to assess lower levels of effect modification. Specifically, based on a simulation study, our investigation had limited power to detect a relative effect modification of 2 but reasonable power to detect a relative effect modification of 3. For some genotypes, including *GSTM1*, *GSTT1*, and *GSTP1*, we found a relative effect modification of approximately 2 in this analysis, so our lack of significance may be a result of power limitations.

### Study limitations

One potential limitation of our study is the use of stationary measures of air pollution to represent personal exposures. Prior research indicates that this exposure misclassification is likely to lead to an underestimation of the health effects of air pollution (Zeger et al. 2000). Several studies, including one conducted in the Greater Boston area, have found that longitudinal measures of ambient PM concentrations are representative of longitudinal variation in personal exposures (Nethery et al. 2008; Rojas-Bracho et al. 2000). Hence, although the use of ambient measurements clearly introduces measurement error into the study, it is not to an unacceptable level. Moreover, Zeger et al. (2000) have shown that most of the measurement error in such cases is Berkson error, which reduces power to find a significant effect but does not bias the estimated effect size. Another potential limitation of our study design was that BP was read only once at each clinical visit, because BP is dynamic and responsive to many stimuli. Furthermore, nonambient PM exposures within or on the way to the clinic were not measured in our study.

Our study population is homogeneous, consisting entirely of elderly men, 97% of whom are white. However, our findings are consistent with studies of PM air pollution conducted in more heterogeneous populations. The Normative Aging Study cohort was not originally recruited to study the effects of air pollution. A number of our participants had only two clinical visits. Although subjects with two measurements obviously contribute less power to the analysis than subjects with three or more measurements, we believe that the study is well powered, with 1,067 total observations for BC and 949 total observations for PM_2.5_ as well as substantial fluctuation in weekly measurements of air pollution over time. Last, we did not correct for multiple comparisons in this analysis. However, we have taken a targeted pathway approach and examined genetic variants known to be associated with oxidative stress.

## Conclusions

More research is needed to address the relation between BC exposure and BP among diverse study populations and to clarify the mechanisms underlying the association between BC and BP. Potential effect modification of the association between BC and BP by genes affecting endothelial function, inflammation, and oxidative stress pathways should be explored in future studies.

Our analysis indicates a positive association between ambient BC concentration and BP. We found no evidence of effect modification of this relation by gene variants related to oxidative stress defense, possibly due to insufficient power, and did not observe an association between PM_2.5_ and BP. These results provide new information to guide future research regarding BC and PM exposures and cardiovascular outcomes.

## Figures and Tables

**Table 1:** Descriptive statistics at baseline for participants with information on BC (*n* = 461) and PM_2.5_ concentrations (*n* = 457).

Study variable	Subjects with BC	Subjects with PM_2.5_
BC (μg/m^3^)^a^,^b^	1.10 ± 0.43	1.06 ± 0.44
PM_2.5_ (μg/m^3^)^a^,^b^	12.15 ± 4.98	12.06 ± 4.93
Age (years)	71.16 ± 6.46	72.18 ± 6.74
Body mass index (kg/m^2^)	28.07 ± 3.89	27.95 ± 3.92
Smoking status [no. (%)]		
Never-smoker	114 (29.53)	116 (28.86)
Former smoker	259 (67.10)	269 (66.92)
Current smoker	13 (3.37)	17 (4.23)
Lifetime smoking (pack-years)	27.38 ± 23.05	28.25 ± 23.77
Alcohol intake (≥ 2 drinks/day) [no. (%)]	76 (19.69)	80 (19.90)
Temperature (°C)^a^	12.18 ± 7.49	11.76 ± 7.85
Relative humidity (%)^a^	68.70 ± 8.74	69.61 ± 8.60
Antihypertensive medication [no. (%)]		
Any	215 (55.70)	243 (60.45)
Calcium channel blockers	45 (11.66)	50 (12.44)
Angiotensin-converting enzyme inhibitors	67 (17.36)	80 (19.90)
Angiotensin receptor agonists	19 (4.92)	22 (5.47)
Beta blockers	114 (29.53)	127 (31.59)
Alpha blockers	46 (11.92)	59 (14.68)
Diuretics	69 (17.88)	81 (20.15)
Statin use [no. (%)]	118 (30.57)	131 (32.59)
Season of clinical visit [no. (%)]		
Spring	90 (23.32)	101 (25.12)
Summer	80 (20.73)	102 (25.37)
Fall	145 (37.56)	119 (29.60)
Winter	71 (18.39)	80 (19.90)
Weekday of clinical visit [no. (%)]		
Monday	22 (5.70)	11 (2.74)
Tuesday	91 (23.58)	103 (25.62)
Wednesday	161 (41.71)	198 (49.25)
Thursday	112 (29.02)	90 (22.39)
Diabetes [no. (%)]	41 (10.62)	47 (11.69)
Race/ethnicity [no. (%)]		
Non-Hispanic white	376 (97.41)	391 (97.26)
Non-Hispanic black	6 (1.55)	7 (1.74)
Hispanic white	3 (0.78)	3 (0.75)
Hispanic black	1 (0.26)	1 (0.25)
Years of education	14.58 ± 2.70	14.65 ± 2.71
Fasting plasma glucose (mmol)	106.97 ± 25.30	106.03 ± 23.85
BP (mmHg)		
SBP	132.44 ± 16.60	131.21 ± 17.26
DBP	77.75 ± 8.96	76.46 ± 9.55

Values are mean ± SD or no. (%).

a Seven-day moving average.

b Mean concentration reported across all study visits rather than just at baseline (1,067 clinical visits for BC, 949 clinical visits for PM_2.5_).

**Table 2:** Mixed linear effects models estimating the change in BP (mmHg) associated with a 1-SD increase^a^ in BC (1,067 visits) and PM_2.5_ (949 visits) levels.^b^

	Change in BP (95% CI)	
Air pollutant	SBP	DBP
BC	1.46 (0.10 to 2.82)*	0.87 (0.15 to 1.59)*
PM_2.5_	0.45 (−0.71 to 1.61)	0.01 (−0.60 to 0.61)

a Corresponding to a 0.43-μg/m^3^ increase in 7-day average BC concentrations and a 4.98-μg/m^3^ increase in 7-day average PM_2.5_ concentrations.

b All regression models are adjusted for age, cigarette smoking, pack-years of smoking, season of clinical visit, weekday of clinical visit, body mass index, diabetes status, statin use, fasting glucose level, 7-day moving average of temperature (linear and quadratic), 7-day moving average of relative humidity (linear and quadratic), hour of clinical visit, year of clinical visit, years of education, race/ethnicity, antihypertensive medication use, and daily alcohol intake.

* *p* < 0.05.

**Table 3:** Modification of the association between a 1-SD increase in BC and BP (mmHg) by SNPs related to antioxidative defense.^a^

	Change in BP (95% CI)	
Genetic variant	SBP	DBP
Catalase C/T (rs480575)		
CC (*n* = 480)	1.27 (−0.77 to 3.30)	0.62 (−0.49 to 1.74)
CT (*n* = 358)	1.36 (−1.16 to 3.89)	0.57 (−0.74 to 1.88)
TT (*n* = 103)	−0.53 (−5.27 to 4.21)	0.59 (−2.12 to 3.29)
*p*-Value for interaction	0.50	0.77

Catalase C1167T (rs769217)		
CC (*n* = 539)	1.40 (−0.52 to 3.32)	0.66 (−0.37 to 1.70)
CT (*n* = 336)	1.49 (−1.05 to 4.04)	0.59 (−0.77 to 1.94)
TT (*n* = 62)	2.51 (−3.90 to 8.92)	0.42 (−3.19 to 4.03)
*p*-Value for interaction	0.37	0.95

Catalase C(–262)T (rs1001179)		
CC (*n* = 594)	1.17 (−0.66 to 3.01)	0.40 (−0.61 to 1.41)
CT (*n* = 289)	0.99 (−1.75 to 3.73)	0.97 (−0.39 to 2.33)
TT (*n* = 50)	1.44 (−8.10 to 10.98)	3.56 (−0.63 to 7.75)
*p*-Value for interaction	0.65	0.42

Catalase A/G (rs2284367)		
AA (*n* = 534)	1.35 (−0.58 to 3.28)	0.68 (−0.36 to 1.72)
AG (*n* = 305)	1.76 (−0.93 to 4.46)	0.67 (−0.72 to 2.06)
GG (*n* = 73)	7.20 (1.46 to 12.93)	2.24 (−1.34 to 5.83)
*p*-Value for interaction	0.61	0.80

Catalase A/G (rs2300181)		
AA (*n* = 498)	2.05 (0.06 to 4.04)	0.64 (−0.40 to 1.68)
AG (*n* = 358)	0.56 (−1.91 to 3.02)	0.38 (−0.87 to 1.64)
GG (*n* = 74)	2.33 (−3.77 to 8.42)	3.59 (−0.15 to 7.32)
*p*-Value for interaction	0.28	0.31

*GSTP1* C2293T (rs1799811)		
AA (*n* = 769)	1.29 (−0.31 to 2.90)	0.47 (−0.38 to 1.32)
AG (*n* = 116)	−0.24 (−5.15 to 4.68)	1.08 (−1.37 to 3.52)
GG (*n* = 5)^b^	—	—
*p*-Value for interaction	0.33	0.66

*GSTP1* A313G (rs1695)		
AA (*n* = 440)	3.20 (0.91 to 5.49)	0.83 (−0.36 to 2.01)
AG (*n* = 363)	−0.53 (−2.77 to 1.70)	0.10 (−1.14 to 1.33)
GG (*n* = 86)	−0.29 (−6.44 to 5.85)	2.48 (−0.26 to 5.21)
*p*-Value for interaction	0.38	0.46

*NQO1* C609T (rs1800566)		
CC (*n* = 680)	1.85 (0.08 to 3.62)	1.10 (0.15 to 2.04)
CT (*n* = 298)	1.43 (−1.13 to 4.00)	0.50 (−0.86 to 1.86)
TT (*n* = 35)	−6.28 (−21.07 to 8.52)	−1.23 (−8.46 to 6.01)
*p*-Value for interaction	0.28	0.16

a All counts reported correspond to number of study center visits. Interaction test results represent interactions between BC and variant allele number only. All regression models are adjusted for age, cigarette smoking, pack-years of smoking, season of clinical visit, weekday of clinical visit, body mass index, diabetes status, statin use, fasting glucose level, 7-day moving average of temperature (linear and quadratic), 7-day moving average of relative humidity (linear and quadratic), hour of clinical visit, year of clinical visit, years of education, race/ethnicity, antihypertensive medication use, and daily alcohol intake.

b Insufficient sample size to obtain sample estimates.

**Table 4:** Effect modification of the association between a 1-SD increase in BC concentrations and BP (mmHg) by gene variants related to oxidative stress.^a^

	Change in BP (95% CI)	
Genetic variant	SBP	DBP
*GSTM1*		
Present (*n* = 494)	0.79 (−1.20 to 2.77)	0.98 (−0.11 to 2.07)
Null (*n* = 527)	1.84 (−0.12 to 3.81)	0.76 (−0.23 to 1.74)
*p*-Value for interaction	0.31	0.84

*GSTT1*		
Present (*n* = 736)	1.26 (−0.38 to 2.91)	0.86 (−0.02 to 1.74)
Null (*n* = 206)	0.56 (−2.59 to 3.71)	−0.09 (−1.44 to 1.62)
*p*-Value for interaction	0.91	0.80

*HMOX-1* microsatellite (GT)_n_ repeat length		
< 25 repeats (*n* = 122)	−0.33 (−4.80 to 4.14)	1.16 (−1.01 to 3.32)
≥ 25 repeats (*n* = 898)	1.81 (0.34 to 3.29)	0.81 (0.02 to 1.61)
*p*-Value for interaction	0.64	0.78

a All counts reported correspond to number of study center visits. Interaction test results represent interactions between BC and variant allele number only. All regression models are adjusted for age, cigarette smoking, pack-years of smoking, season of clinical visit, weekday of clinical visit, body mass index, diabetes status, statin use, fasting glucose level, 7-day moving average of temperature (linear and quadratic), 7-day moving average of relative humidity (linear and quadratic), hour of clinical visit, year of clinical visit, years of education, race/ethnicity, antihypertensive medication use, and daily alcohol intake.
